# Lowering the setting value of the esophageal endoscopic submucosal dissection device enabled tissue damage control in vitro porcine model

**DOI:** 10.1038/s41598-022-06533-9

**Published:** 2022-02-23

**Authors:** Yukiko Yamaguchi, Masaya Uesato, Shohei Yonemoto, Tetsuro Maruyama, Ryuma Urahama, Hiroshi Suito, Takashi Kishimoto, Yuki Shiko, Yoshihito Ozawa, Yohei Kawasaki, Hisahiro Matsubara

**Affiliations:** 1 Department of Frontier Surgery, Chiba University Graduate School of Medicine, Chiba, 260-8670 Japan; 2 Department of Molecular Pathology, Chiba University Graduate School of Medicine, Chiba, 260-8670 Japan; 3 Biostatistics Section, Clinical Research Data Center, Chiba University Hospital, Chiba, 260-8670 Japan

**Keywords:** Cancer, Gastroenterology

## Abstract

One of the complications of esophageal endoscopic submucosal dissection (ESD) is postoperative stricture formation. Stenosis formation is associated with inflammation and fibrosis in the healing process. We hypothesized that the degree of thermal damage caused by the device is related to stricture formation. We aimed to reveal the relationship between thermal damage and setting value of the device. We energized a resected porcine esophagus using the ESD device (Flush Knife 1.5). We performed 10 energization points for 1 s, 3 s, and 5 s at four setting values of the device. We measured the amount of current flowing to the conducted points and the temperature and evaluated the effects of thermal damage pathologically. As results, the mean highest temperatures for 1 s were I (SWIFT Effect3 Wat20): 61.19 °C, II (SWIFT Effect3 Wat30): 77.28 °C, III (SWIFT Effect4 Wat20): 94.50 °C, and IV (SWIFT Effect4 Wat30): 94.29 °C. The mean heat denaturation areas were I: 0.84 mm^2^, II: 1.00 mm^2^, III: 1.91 mm^2^, and IV: 1.54 mm^2^. The mean highest temperature and mean heat denaturation area were significantly correlated (*P* < 0.001). In conclusion, Low-current ESD can suppress the actual temperature and thermal damage in the ESD wound.

## Introduction

The use of endoscopic submucosal dissection (ESD) as a safe procedure for early esophageal cancer is increasing worldwide^1, 2^. A subacute complication of ESD is postoperative stricture formation, which can cause severe food passage disorder^3^. Widespread excision of large lesions is a widely known risk factor of postoperative stenosis^1–5^. However, excision of large lesions cannot always be avoided. There are reports that systemic and local steroid administration prevents postoperative stenosis^6–8^. However, these procedures involve risks, such as infection and perforation^9, 10^, and cannot completely prevent stenosis^2, 8^. There are other innovative methods, such as transplantation of cell sheets^11^, collagen patches^12^, and polyglycolic acid sheets^13^, but there are high barriers to their practical use in terms of cost, simplicity, and safety^14^. The log bridge method has been proposed for subcircumferential lesions^15^, but it is difficult to preserve the mucosa with circumferential lesions.

Using a dog model, Honda et al.^16^ showed that stricture formation was associated with inflammation in the ESD wound and fibrosis in the healing process. We speculated that the degree of thermal damage caused by the ESD device was related to inflammation and fibrosis. We therefore focused on the heat generated in the tissue during ESD and the amount of current flowing to the wound.

There is no standard setting value for high-frequency output devices, and endoscopists mainly rely on personal or reported experience^17^. Most endoscopic physicians determine the setting value according to their own preferences so that the device can be easily operated during ESD. We aimed to show that the amount of current flowing to the conducted points, the temperature, and the thermal damage to tissue vary depending on the setting value of the high-frequency output device during ESD, and to propose the optimal setting value that has minimal effect on the wound.

## Materials and methods

### Experimental procedure

**Figure 1:**
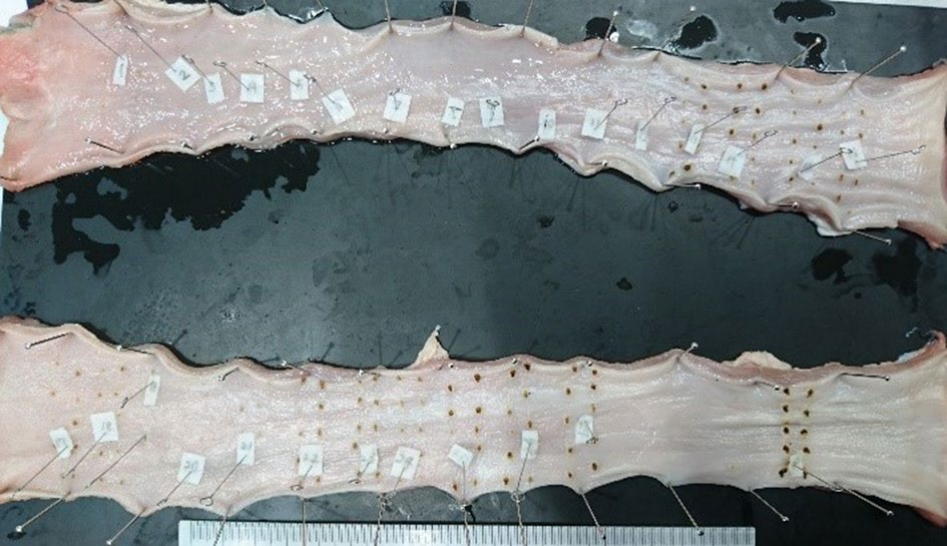
The resected porcine esophagus was pinned onto the rubber plate. Energization was repeated 10 times under the same conditions.

The study was performed in Chiba University Hospital. The experimental sample was obtained by purchasing the esophagus of two porcines that had already been removed. This sample was selected because the tissue is close to the human body, and the number is kept to a minimum. All procedures were approved by the institutional animal care and use committee of the Chiba University, in compliance with the national guideline of Japan. The equipment consisted of a microammeter (Power Meter 3335/HIOKI E.E. CORPORATION, Japan), thermography (Thermo FLEX F50/Nippon Avionics Co., Ltd. Japan), high-frequency energy output device (VIO200D/Erbe Elektromedizin GmbH, Germany), and ESD device (Flush Knife 1.5 mm, DK2620JN15S; Fujifilm Medical, Tokyo, Japan) that we usually use for treatment. The microammeter was used to measure the energized current (mA) and the thermograph to measure the temperature (°C). The camera of thermography was set 10 cm from the porcine esophageal mucosa. We energized the sample in vitro with the Flush Knife. The tip of the device contacted with the sample without pressing. Energization was performed at four setting values (I: SWIFT COAG mode, Effect 3, Wat 20/II: SWIFT COAG mode, Effect 3, Wat 30/III: SWIFT COAG mode, Effect 4, Wat 20/IV: SWIFT COAG mode, Effect 4, Wat 30 (Table 1) of the high-frequency output device. The sample was energized for 1 s, 3 s, and 5 s for 10 repetitions under the same conditions (Fig. 1).

**Table 1:** Setting value of high frequency energy output device.

Setting value	Effect	Wattage
I	3	20
II	3	30
III	4	20
IV	4	30

### Assessment of thermal damage

**Figure 2:**
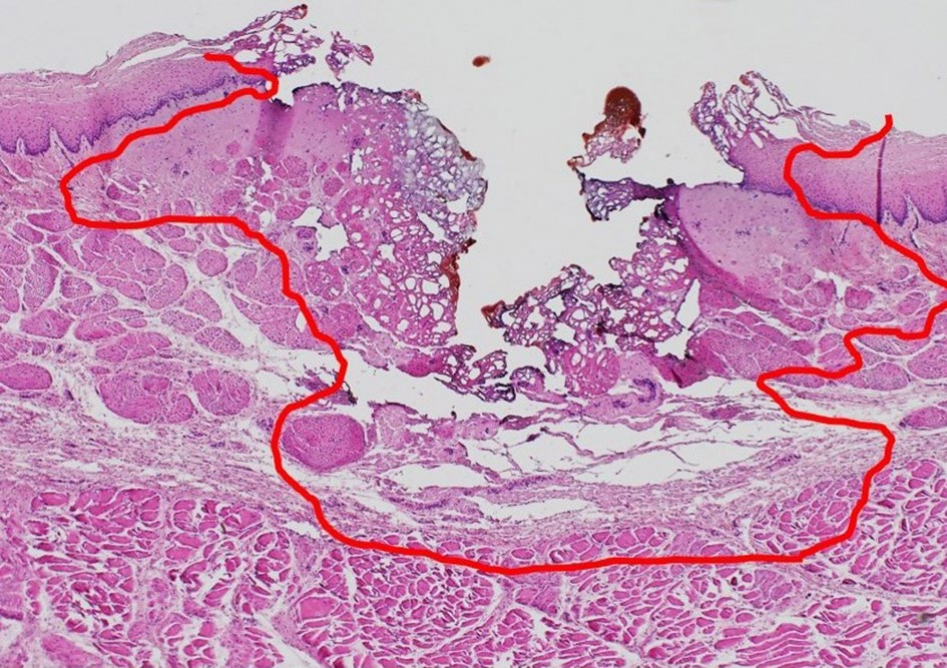
The conduction point was excised in the direction of the minor axis of the porcine esophagus and stained with hematoxylin and eosin. One pathologist determined the extent of heat denaturation.

After the experiment, the esophagus was preserved in formalin. The conduction points were then excised in the direction of the minor axis of the esophagus and stained with hematoxylin and eosin. One pathologist determined the extent of heat denaturation (Fig. 2) and measured the area of thermal damage using the GNU Image Manipulation Program.

### Statistical analysis

The data are expressed as means in the text and as means ± standard deviation in the figures. The associations between the setting values of the high-frequency output device and the integrated energization amount (mA), the maximum energization amount (mA), the maximum temperature (°C), the integrated temperature (°C), the vertical distance of heat denaturation (µm), and the heat denaturation area (mm^2^) were tested using Spearman’s rank correlation coefficient, Pearson’s correlation coefficient, and analysis of variance (ANOVA) with contrast. We also performed linear regression with the integrated energization amount as the independent variable and the cumulative temperature as the dependent variable. To assess the fitting of this model, we calculated the *R*^2^ value. A two-trailed *P* value < 0.05 was considered to indicate statistical significance. Statistical analyses were performed with SAS for Windows (Ver. 9.4, SAS Institute Inc., Cary, NC, USA).

## Results

**Figure 3:**
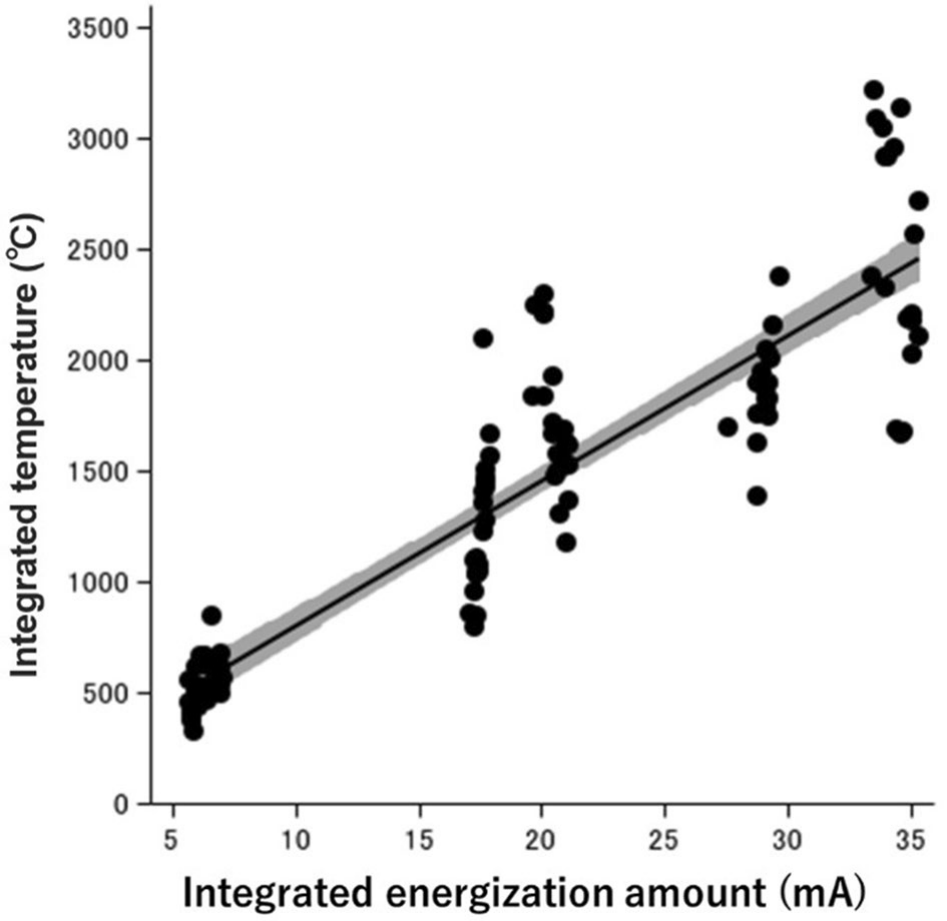
Scatter plot and regression line showing the correlation between integrated energization and temperature (Pearson’s correlation coefficient; r = 0.91). The regression follows (Integrated temperature = 164.40 + 82.74 × (integrated energization amount); *R*^2^ = 0.82).

Analysis was performed of 10 energization points under 12 conditions in the porcine esophagus. Energization was performed for 1 s, 3 s, and 5 s at four setting values (Table 1). The outcomes of the study are summarized in Table 2 and illustrated in Fig. 3.

**Table 2:** Measurement results and statistical analysis results under each setting.

Energized condition^a^	I	II	III	IV	r (p-value)^b)^	I vs IV (p-value)^c^
**Energized for 1 s**						
Maximum energization amount (mA)	1.19 (0.03)	1.30 (0.08)	1.40 (0.05)	1.46 (0.09)	0.84 (< 0.001)	< 0.001
Integrated energization amount (mA)	4.40 (0.05)	4.59 (0.10)	4.98 (0.37)	5.48 (0.12)	0.87 (< 0.001)	< 0.001
Maximum temperature (°C)	61.19 (8.19)	77.28 (6.40)	94.50 (10.85)	94.29 (16.69)	0.79 (< 0.001)	< 0.001
Integrated temperature (°C)	403.21 (34.36)	478.70 (31.84)	612.60 (64.04)	608.28 (98.66)	0.81 (< 0.001)	< 0.001
Heat denaturation vertical distance (μm)	560.18 (183.28)	587.73 (218.80)	1117.78 (370.63)	741.24 (217.29)	0.43 (0.005)	0.125
Heat denaturation area (mm^2^)	0.84 (0.23)	1.00 (0.36)	1.91 (0.47)	1.54 (0.55)	0.63 (< 0.001)	< 0.001
**Energized for 3 s**						
Maximum energization amount (mA)	1.21 (0.04)	1.34 (0.06)	1.47 (0.04)	1.50 (0.04)	0.90 (< 0.001)	< 0.001
Integrated energization amount (mA)	13.08 (0.38)	13.59 (0.11)	16.24 (1.04)	16.33 (0.47)	0.84 (< 0.001)	< 0.001
Maximum temperature (°C)	59.35 (7.41)	88.26 (10.29)	94.52 (8.72)	100.20 (18.71)	0.71 (< 0.001)	< 0.001
Integrated temperature (°C)	990.94 (112.72)	1439.86 (130.33)	1630.25 (209.27)	1838.34 (406.93)	0.80 (< 0.001)	< 0.001
Heat denaturation vertical distance (μm)	482.55 (221.08)	670.40 (101.18)	1126.14 (644.76)	1035.12 (483.72)	0.50 (0.001)	0.006
Heat denaturation area (mm^2^)	0.86 (0.31)	1.34 (0.25)	2.29 (1.15)	2.15 (1.07)	0.55 (< 0.001)	0.002
**Energized for 5 s**						
Maximum energization amount (mA)	1.26 (0.03)	1.29 (0.07)	1.48 (0.03)	1.45 (0.06)	0.77 (< 0.001)	< 0.001
Integrated energization amount (mA)	22.03 (0.48)	21.33 (2.86)	28.03 (0.36)	27.14 (0.47)	0.70 (< 0.001)	< 0.001
Maximum temperature (°C)	72.95 (10.02)	73.90 (12.79)	86.99 (12.93)	85.64 (13.57)	0.54 (0.000)	0.028
Integrated temperature (°C)	1841.81 (78.38)	1916.41 (279.87)	2379.52 (453.24)	2494.51 (632.67)	0.47 (0.002)	0.001
Heat denaturation vertical distance (μm)	564.05 (156.06)	586.98 (274.26)	944.80 (334.46)	1006.68 (459.02)	0.48 (0.002)	0.005
Heat denaturation area (mm^2^)	0.90 (0.38)	0.98 (0.57)	2.25 (1.01)	2.26 (1.12)	0.60 (< 0.001)	0.001

^a^I: Swift coagulation mode, Effect 3, Wat 20; II: Swift coagulation mode, Effect 3, Wat 30; III: Swift coagulation mode, Effect 4, Wat 20; IV: Swift coagulation mode, Effect 4, Wat 30.

^b^Spearman's correlation with p-value.

^c^Anova with specific contrast (I vs IV).

### Amount of energization

The mean current peak values were I: 1.19/1.21/1.26 (mA), II: 1.30/1.34/1.29 (mA), III: 1.40/1.47/1.48 (mA), and IV: 1.46/1.50/1.45 (mA). A significant correlation was observed between the current peak values and the setting conditions (*r* = 0.84, *P* < 0.001/*r* = 0.90, *P* < 0.001/*r* = 0.77, *P* < 0.001). The current peak values of I were significantly lower than those of IV (*P* < 0.001/*P* < 0.001/*P* < 0.001). The integrated energization amount was I: 4.40/13.08/22.03 (mA), II: 4.59/13.59/21.33 (mA), III: 4.98/16.24/28.03 (mA), and IV: 5.48/16.33/27.14 (mA), and a significant correlation was observed between the amount of integrated energization and the setting conditions (*r* = 0.87, *P* < 0.001/*r* = 0.84, *P* < 0.001/*r* = 0.70, *P* < 0.001). The amount of integrated energization of I was significantly lower than that of IV (*P* < 0.001/*P* < 0.001/*P* < 0.001).

### Temperature

The mean highest temperatures at 1 s/3 s/5 s for each setting were as follows: I: 61.19/59.35/72.95 (°C), II: 77.28/88.26/73.90 (°C), III: 94.50/94.52/86.99 (°C), and IV: 94.29/100.2/85.64 (°C), and a significant correlation was observed between the highest temperature and the setting conditions (*r* = 0.79, *P* < 0.001/*r* = 0.71, *P* < 0.001/*r* = 0.54, *P* < 0.001). The highest temperature of I was significantly lower than that of IV (*P* < 0.001/*P* < 0.001/*P* = 0.028). The mean accumulated temperatures were I: 403.21/990.94/1841.81 (°C), II: 478.7/1439.86/1916.41 (°C), III: 612.6/1630.25/2379.52 (°C), and IV: 608.3/1838.34/2494.51 (°C), and a significant correlation was observed between the accumulated temperature and the setting conditions (*r* = 0.81, *P* < 0.001/*r* = 0.80, *P* < 0.001/*r* = 0.47, *P* = 0.002). The accumulated temperature of I was significantly lower than that of IV (*P* < 0.001/*P* < 0.001/*P* = 0.001).

**Figure 4:**
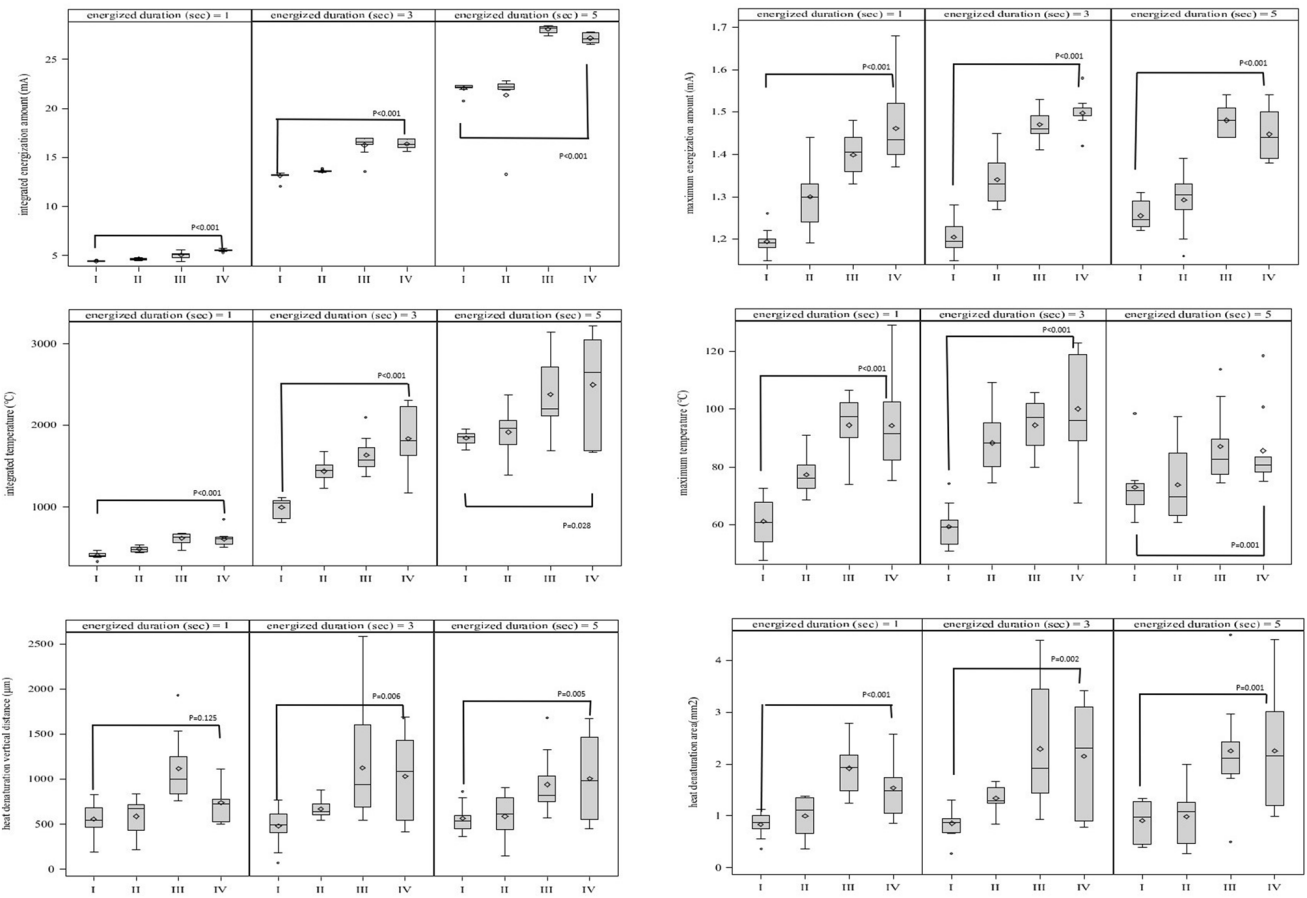
Measurement results and statistical analysis results under each setting.

The integrated temperature of the tissue was increased by increasing the accumulated energization amount (*r* = 0.91, *P* < 0.001). A significantly strong correlation between the accumulated energization amount and the accumulated temperature was found, and a prediction calculation formula for the accumulated temperature was derived: (Integrated temperature = 164.40 + 82.74 × (integrated energization amount); *R*^2^ = 0.82) (Fig. 4).

### Assessment of thermal damage

The mean vertical distance of thermal damage was I: 560.18/482.55/564.05 (µm), II: 587.73/670.40/586.98 (µm), III: 1117.78/1126.14/944.80 (µm), and IV: 741.24/1035.12/1006.68 (µm), and a significant correlation was observed between the vertical distance of thermal damage and the setting conditions (*r* = 0.43, *P* = 0.005/*r* = 0.50, *P* = 0.001/*r* = 0.48, *P* = 0.002). There was no significant difference between the vertical distance of thermal damage of I and IV under 1 s of energization (*P* = 0.125/*P* = 0.006/*P* = 0.005). The mean area of heat denaturation was I: 0.84/0.86/0.90 (mm^2^), II: 1.00/1.34/0.98 (mm^2^), III: 1.91/2.29/2.25 (mm^2^), and IV: 1.54/2.15/2.26 (mm^2^), and a significant correlation was observed between the area of heat denaturation and the setting conditions (*r* = 0.63, *P* < 0.001/*r* = 0.55, *P* < 0.001/*r* = 0.60, *P* < 0.001). The area of heat denaturation of IV was significantly larger than that of I (*P* < 0.001/*P* = 0.002/*P* = 0.001).

## Discussion

The usefulness of steroid administration for the prevention of stenosis after esophageal ESD has been reported. This is because local inflammation and fibrosis that cause stenosis can be prevented by steroids^5^. Nevertheless, there have never been surgical procedures that do not cause inflammation or fibrosis. This study compared the effects of the setting values of the high-frequency output device by measuring the amount of current flowing to the conducted points, the temperature, and the thermal damage to the tissue. We evaluated the degree of thermal damage at the energization point pathologically and found that the lower the setting value, the less the thermal damage.

When performing ESD, we use different modes of high-frequency energy output devices, depending on the situation^16^. Various parameters, including power settings, allow control of tissue heating and the resulting outcomes of cutting and/or coagulation of tissue^17^. For the surrounding incision, we use the ENDO CUT I mode, which enables us to incise using sudden heat, which causes the water in the tissue to evaporate and the cell membrane to break due to water vapor. This mode provides both cutting and coagulation effects. The SWIFT COAG mode, which allows incision with hemostasis, is used for exfoliation of the submucosa. This mode uses higher voltage and lower current, providing a smaller cutting effect and a larger hemostatic effect^18^. In cases of bleeding, we use a SOFT COAG mode so that the moisture in the tissue evaporates and contracts to cause a hemostatic effect, resulting in coagulation. These modes are adjusted by effects and watts. The effect settings range from 1 to 8. If the effect setting is high, the voltage rises and the current flows quickly to the tissue, so that coagulation is completed quickly^19^. On the other hand, the lower the effect, the lower the voltage, and the current flows through the tissue relatively slowly.

During the ESD procedure, it takes the longest time to dissect the submucosa using the SWIFT COAG mode. Tonai et al.^20^ reported that there are two basic patterns of electrocautery using the high-frequency energy output device. One is cut current, and the other is coagulation current. Coagulation current has a higher peak voltage than cut current, and thus theoretically can cause more severe thermal damage to the tissue than cut current^18, 20^. The authors performed esophageal ESD on a porcine model with a various single mode and showed that the ENDO CUT mode resulted in less inflammation of the tissue than the COAG mode. However, in general, COAG mode is used for actual ESD during the exfoliation of the submucosa. In contrast, the authors did not mention the heat actually generated at the wound. Therefore, we focused on the SWIFT COAG mode whose impact on the wound is considered to be the largest, and measured the actual heat generated at the wound. No study has measured the heat generated from the ESD device using thermography.

In this study, we measured the peak current and the integrated current flowing to the tissue and the highest temperature and the integrated temperature during energizing with the ESD device at various setting values. “Effect” signifies the voltage force that pushes the current, and the output setting of the high-frequency energy output device is controlled by the effect value and the maximum wattage value. When the wattage value was lowered under the condition of Effect 3, all measured values were lowered. On the other hand, under the condition of Effect 4, the measured values changed little, even if the wattage value was changed. Rather, lowering the wattage value resulted in higher temperatures and greater thermal damage under Effect 4. In general, the Effect 3 condition had less effect on the wound than the Effect 4 condition. This suggests that the value of Effect in the high-frequency energy output device is most likely related to the heat generated at the ESD wound.

We have shown positive correlations between the amount of current flowing to the tissue during ESD, the temperature of the device touching the tissue, and thermal damage to the tissue. The heat generated at the wound decreased when the setting value was lowered. There was a strong correlation between the amount of current consumed and the heat generated at the wound. We also evaluated the degree of thermal damage at the energization point pathologically and found that the lower the setting value, the less the thermal damage. Therefore, low-current ESD can contribute to the suppression of the local damage in ESD wound. The suppression of local thermal damage may contribute toward the suppression of the local inflammation and may result in the alleviation of postoperative stenosis.

There are limitations to this study. The microammeter measured the current consumed by the high-frequency output device. We considered that the actual current flowing locally could be approximated by subtracting the standby current from the working current during ESD. The study used a resected porcine esophagus, which may differ from human esophageal tissue^21^. Since there is no thermography that can be used in the esophagus of a living porcine, we conducted this experiment using the extracted esophagus of porcine. In the actual ESD procedure, submucosal injection is performed before energization. However, if local injection was performed in this experiment, the conditions would change for each energization point. Therefore, local injection was not performed. Above all, the current status of energization is different from the actual ESD situation. Further research is needed to clarify the relationship between the setting value of the high-frequency energy output device and postoperative stenosis. Studies should compare the degree of postoperative stenosis with the use of esophageal ESD under a standard setting value and a lower setting value. In addition, it will be necessary to verify whether ESD can be performed safely at a low setting value without losing sharpness and hemostatic effect. We are presently conducting the clinical trial “The association between quantity of electricity during ESD and postoperative esophageal stricture formation: a randomized, double-blind, parallel-group trial”. So far, the results show that the ESD procedure can be performed safely with a low setting value similar to that with usual setting.
